# Two-Year Changes in Hyperuricemia and Risk of Diabetes: A Five-Year Prospective Cohort Study

**DOI:** 10.1155/2018/6905720

**Published:** 2018-12-30

**Authors:** Jia Liu, Lixin Tao, Zhan Zhao, Yongmin Mu, Dechun Zou, Jingbo Zhang, Xiuhua Guo

**Affiliations:** ^1^School of Public Health, Capital Medical University, Beijing, China; ^2^Beijing Municipal Key Laboratory of Clinical Epidemiology, Beijing, China; ^3^State Key Lab of Transducer Technology, Institute of Electronics, Chinese Academy of Sciences, Beijing, China; ^4^University of Chinese Academy of Sciences, Beijing, China; ^5^Computer Department, Beijing Information Science and Technology University, Beijing, China; ^6^Beijing National Laboratory for Molecular Sciences, College of Chemistry and Molecular Engineering, Peking University, Beijing, China; ^7^Department of Information, Beijing Physical Examination Center, Beijing, China

## Abstract

**Background:**

Hyperuricemia is known to be a risk factor for diabetes. However, information is limited regarding the association between changes in hyperuricemia and the risk of diabetes.

**Methods:**

A total of 15,403 participants who were free of diabetes at the time of 2009 and 2011 surveys in the Beijing Health Management Cohort (BHMC) study were recruited and followed up until 2016. Participants were classified into four groups according to 2-year changes in hyperuricemia: no hyperuricemia, remittent hyperuricemia, incident hyperuricemia, and persistent hyperuricemia. Modified Poisson regression models were used to evaluate the effect of 2-year changes in hyperuricemia on the risk of diabetes.

**Results:**

During the 5-year follow-up, we identified 841 new cases of diabetes (216 women). Remittent hyperuricemia and incident hyperuricemia had a 35% and 48% higher risk for developing diabetes compared with no hyperuricemia. Especially, persistent hyperuricemia was associated with a 75% higher risk of diabetes (RR = 1.75, 95% CI = 1.47-2.08). Compared with minor serum uric acid (SUA) change, over 10% decline and over 30% increase in SUA levels were subsequently associated with lower (RR = 0.84, 95% CI = 0.72-0.99) and higher (RR = 1.71, 95% CI = 1.27-2.30) diabetes risk, respectively.

**Conclusions:**

Changes in hyperuricemia, especially persistent hyperuricemia, are more appropriate to reflect the risk of diabetes than a single measurement of hyperuricemia at baseline. Strategies aiming at preventing hyperuricemia are urgently needed to reduce the increasing burden of diabetes.

## 1. Introduction

Diabetes is an increasingly important metabolic disorder globally [1]. In 2017, 451 million adults are estimated to have diabetes, and this number is expected to rise to 693 million by 2045 worldwide [2]. Diabetes is also a known risk factor for cardiovascular disease and premature death [3, 4]. Thus, the growing long-term disease burden of diabetes indicates that prevention is urgently needed, which, in turn, highlights the necessity of early detection on modifiable risk factors.

Serum uric acid (SUA) is the final breakdown product of purine metabolism. Abnormalities in uric acid metabolism result in hyperuricemia [5–7]. Emerging evidence suggests that hyperuricemia is a novel risk factor for the development of diabetes [8–11]. The Rotterdam Study demonstrated that SUA at baseline was a strong and independent risk factor for diabetes [8]. The Framingham Heart Study, which was conducted in two generations, suggested that individuals with higher uric acid were at a higher future risk of type 2 diabetes [9]. In addition, a retrospective cohort study in America showed that hyperuricemia was associated with excess risk for developing diabetes [10]. Data from the Atherosclerosis Risk in Communities Study also found that uric acid level was associated with an increased risk of diabetes after adjustment for other risk factors [11].

However, majority of previous studies were based on baseline SUA at a single time point, failing to take into account the potential effect of changes in hyperuricemia in follow-up. This could result in biased estimation of the relationship between hyperuricemia and diabetes. Furthermore, there is limited information about how SUA changes within individuals over time. Both with respect to improving stratification of diabetes risk in populations and providing clues for exploring the potential effects of SUA lowering on diabetes, perspective studies that evaluate the impact of changes of hyperuricemia on the risk of diabetes are essential.

## 2. Materials and Methods

### 2.1. Study Population

**Figure 1:**
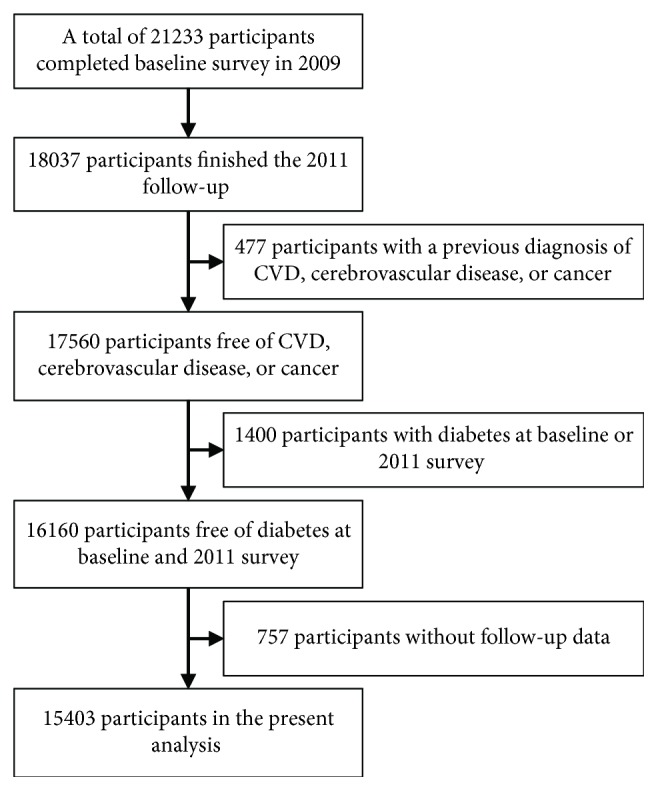
Study population selection flowchart in the Beijing Health Management Cohort, 2009–2016.

The Beijing Health Management Cohort (BHMC) study is a large longitudinal cohort study that investigates the development of metabolic disorders in healthy individuals from urban areas of northeast China. The current and retired employees in fixed work environments in Beijing have been followed with data from a physical examination, a general health questionnaire, and laboratory measurements collected at every examination [12]. A total of 21233 individuals were recruited to participate in this study in 2009. Among these individuals, 15403 participants who were free of diabetes, cardiovascular disease, and cancer were included in the final analysis (Figure 1). In this study, the 2012 survey of the BHMC study was used as the starting point of the follow-up, and the 2016 survey was used as the end point of the follow-up.

Ethical approval for the study was obtained from the Ethics Committee of the Capital Medical University (number 2013SY26), and all procedures were conducted according to the 1964 Declaration of Helsinki and its later amendments. All participants were required to provide a written informed consent.

### 2.2. Data Collection

For each participant, the SUA levels and potential covariates for diabetes were collected by trained examiners at baseline and during follow-up. Height and weight were measured when participants wore lightweight clothing and took off shoes. Body mass index (BMI) was calculated to measure overall obesity. Participants were asked to avoid smoking or caffeine intake during 30 minutes before the measurements of blood pressure. After sitting down to rest for 5 minutes, systolic blood pressure (SBP) and diastolic blood pressure (DBP) were measured in the right arm of the sitting subject using an electronic sphygmomanometer. Blood pressure measurements were recorded three times at 1 to 3-minute intervals, and the average of the second and the third measurements was used in the data analysis.

Laboratory tests included SUA, fasting plasma glucose (FPG), total cholesterol (TC), high-density lipoprotein cholesterol (HDLC), and triglycerides (TG). Blood samples were taken from participants after a 12-hour overnight fast. Enzymatic methods were used to measure SUA, FPG, TC, HDLC, and TG on a chemistry analyzer (Beckman LX 20, America) at the hospital's central laboratory. All analyses were performed according to the manufacturer's recommendations. Family history of diabetes and information on lifestyle factors, including educational level, physical activity, smoking status, and alcohol intake status were obtained from the health questionnaire. Physical activity level was classified as low, moderate, or high. Smoking status was assessed by reporting to have smoked ≥100 cigarettes during lifetime. Drinking status was assessed by reporting to have consumed alcohol ≥12 times in the preceding year.

### 2.3. Definition of Hyperuricemia and Changes in Hyperuricemia

Hyperuricemia was defined as SUA level greater than 7 mg/dL in males and SUA level greater than 6 mg/dL in females [13]. In addition, we conducted sensitivity analysis in which hyperuricemia was defined as SUA level greater than 6.8 mg/dL [14].

Four hyperuricemia groups were defined according to changes in hyperuricemia from baseline in 2009 to the follow-up survey in 2011. No hyperuricemia was defined as hyperuricemia absent both at the baseline and at the follow-up survey. Remittent hyperuricemia was defined as hyperuricemia that was present only at the baseline survey. Incident hyperuricemia was defined as hyperuricemia that was present only at the follow-up survey. Persistent hyperuricemia was defined as hyperuricemia present both at the baseline and at the follow-up survey. No hyperuricemia was set as the reference group [15].

### 2.4. Definition of Diabetes

According to recommendations from American Diabetes Association, diabetes was defined as a fasting plasma glucose level ≥ 7.0 mmol/L or with the use of antidiabetic medication [16]. The glucose hexokinase method was used to measure fasting blood samples.

### 2.5. Statistical Analyses

Data were presented as the mean ± standard deviation for continuous variables and percentages for categorical variables. The Kruskal-Wallis tests or chi-square tests were used to compare the baseline characteristics of the participants among four hyperuricemia groups. In addition, we categorized participants based on percentage SUA change into four groups: ≤−10%, >−10% and ≤10%, >10% and ≤30%, and >30%. A minor change of SUA (>−10% and ≤10%) was considered to be the reference group.

Modified Poisson regression analyses were applied to estimate the relative risk (RR) and 95% confidence interval (CI) of diabetes by the changes in hyperuricemia [17]. All potential confounding variables in the regression analyses were collected at baseline in 2009. Model 1 was adjusted for age and sex, and then model 2 was additionally adjusted for educational level, physical activity, smoking status, alcohol intake status, and family history of diabetes at baseline. Lastly, model 3, which was based on model 2, was additionally adjusted for SBP, DBP, TC, TG, and HDLC at baseline.

All statistical analyses were performed using SAS software version 9.2 (SAS Institute, Chicago, IL, USA), and *P* < 0.05 was considered statistically significant.

## 3. Results

A total of 15403 eligible participants were included in the analysis in this study. According to changes in hyperuricemia category, the baseline characteristics of the study population are presented in Table 1. Age, sex, BMI, SBP, DBP, TC, TG, HDLC, physical activity, smoking, alcohol consumption, and family history of diabetes at baseline were significantly associated with changes in hyperuricemia from 2009 to 2011 (*P* < 0.05).

After the 5-year follow-up, 841 diabetes events were identified (625 men and 216 women). The prevalence rate of diabetes increased from 3.93% in the no hyperuricemia group to 13.45% in the persistent hyperuricemia group. The adjusted RRs and 95% CIs of incident diabetes associated with changes in hyperuricemia are presented in Table 2. In the final multivariable Poisson model, compared with participants in the no hyperuricemia group, participants in the remittent hyperuricemia group and the incident hyperuricemia group had a 1.35-fold and 1.48-fold greater risk for developing diabetes. Especially, the risk of diabetes became the highest in the persistent hyperuricemia group using no hyperuricemia as the reference group after adjusting for other potential confounding factors (RR = 1.75, 95% CI = 1.47-2.08). In sensitivity analysis, the association between changes in hyperuricemia and diabetes was consistent when hyperuricemia was defined as SUA level greater than 6.8 mg/dL (Table 3).

**Figure 2:**
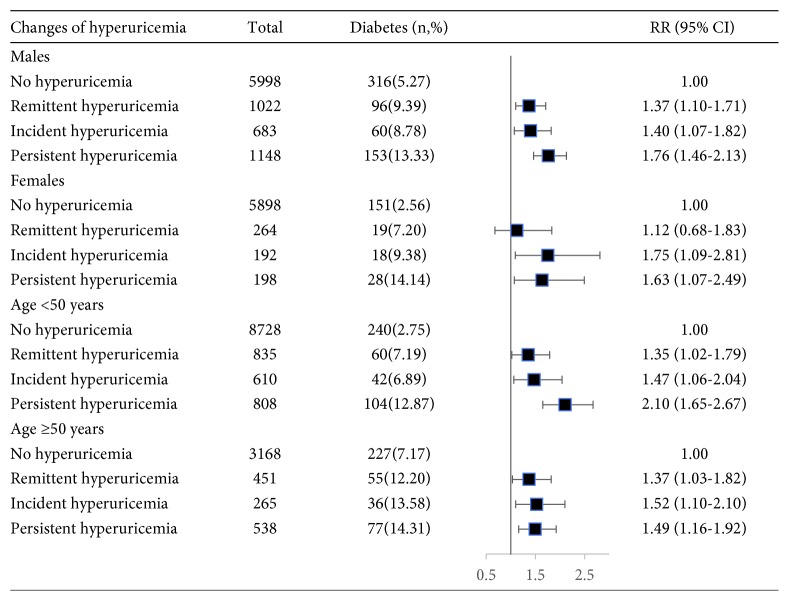
The association between changes of hyperuricemia and incident diabetes stratified by age and sex. Data were relative risks (RRs) and 95% confidence intervals (CIs). Adjusted for age, sex, education level, smoking, alcohol consumption, physical activity, family history of diabetes, body mass index, systolic blood pressure, diastolic blood pressure, total cholesterol, triglycerides, and high-density lipoprotein cholesterol at baseline.

The associations between changes in hyperuricemia and diabetes stratified by age and sex are presented in Figure 2. Compared with participants with no hyperuricemia, participants with persistent hyperuricemia showed significant RRs for developing diabetes in males (RR = 1.76, 95% CI = 1.46-2.13) and in females (RR = 1.63, 95% CI = 1.07-2.49) and in participants younger than 50 years (RR = 2.10, 95% CI = 1.65-2.67) and in participants at least 50 years (RR = 1.49, 95% CI = 1.16-1.92) after adjustment for potential confounders.

We investigated the association between the percentage change of SUA and the risk of diabetes (Table 4). After the 2-year follow-up, 26.8% of study participants showed a percentage SUA loss of ≥10% and 23.4% of participants showed a percentage SUA gain of ≥10%. Compared with a percentage SUA change of −10% to 10%, participants with a percentage SUA gain of >30% had a 71% higher risk of diabetes (RR = 1.71, 95% CI = 1.27-2.30), and participants with a percentage SUA loss of ≥10% had a 16% lower risk of diabetes (RR = 0.84, 95% CI = 0.72-0.99) after adjusting for potential confounders. Significant positive linear trends in the risk of diabetes with increasing SUA were observed in all three models (*P* for trend < 0.05 in all).

In addition, the risk of diabetes associated with baseline hyperuricemia was assessed (Table 5). After adjusting for potential confounders, participants with hyperuricemia at baseline showed a significant 48% higher risk for developing diabetes compared with participants with no hyperuricemia (RR = 1.48, 95% CI = 1.28-1.71).

## 4. Discussion

The current study links the development of diabetes and the 2-year changes of hyperuricemia in Chinese urban adults after adjusting for other known major diabetic risk factors. The remittent, incident, and persistent hyperuricemia were significantly associated with the increased risk of developing diabetes. Furthermore, the persistent presence of hyperuricemia had the highest risk of diabetes compared with the remittent or incident hyperuricemia.

The Framingham Heart Study, based on 4883 original and 4292 offspring individuals, reported that multivariable relative risks per mg/dL increase in SUA levels for developing diabetes were 1.20 for the original cohort and 1.15 for the offspring cohort [9]. Similarly, the Rotterdam study on 4536 participants found that individuals with the top quartile (SUA > 6.2 mg/dL) had a 1.68-fold greater risk of incident type 2 diabetes using the lowest quartile (SUA ≤ 4.5 mg/dL) as the reference group[8]. Relying on mean SUA levels, a retrospective cohort study in America also presented a significant association between hyperuricemia and risk of new onset diabetes among male veterans with gout [10]. In a meta-analysis of eight prospective cohort studies including a total of 32016 participants, the highest category of SUA level showed a combined RR of 1.56 for developing type 2 diabetes compared with the lowest category of SUA level [18].

In our study, baseline hyperuricemia was associated with a 48% increased risk of diabetes, which was in accordance with the above studies [8–11, 18, 19]. However, previous studies were generally limited to baseline SUA level, regardless of the potential effect of changes in hyperuricemia over time. Our findings substantially extended the evidence from a previous study by demonstrating that 2-year changes of hyperuricemia were associated with the altered risk of diabetes. Particularly, participants who had persistent presence of hyperuricemia, as compared with participants with the remittent or incident hyperuricemia, were at a greater risk of diabetes. Moreover, high prevalence of hyperuricemia and diabetes places an alarmingly heavy burden on patients and health care systems [20–22]. Our results provide prospective confirmation of the predictive ability of hyperuricemia changes for diabetes. Therefore, more attention should be paid to the individuals with hyperuricemia, especially persistent hyperuricemia in the clinical guideline for the prevention of diabetes. Currently, asymptomatic hyperuricemia is not considered an indication for treatment with uric acid-lowering drugs [23]. Large randomized controlled trials are therefore warranted to assess the benefit and the risk bringing with uric acid-lowering treatment in patients with asymptomatic hyperuricemia.

The potential biological mechanisms underlying this association have not yet been fully elucidated. Evidence indicates that an over accumulation of uric acids can lead to endothelial dysfunction through nitric oxide degeneration or activation of the renin-angiotensin system, which results in insulin resistance and thus diabetes [24, 25]. Additionally, hyperuricemia has been reported to associate with oxidative stress, which contributes to the development of type 2 diabetes [26, 27]. These findings from experimental studies support hyperuricemia as a causal factor of diabetes.

There is accumulating evidence to support the association between elevated SUA and future risk of diabetes [18, 28]. Nevertheless, whether a reduction in SUA subsequently leads to a lower risk of diabetes remains uncertain. A few clinical trials provided encouraging evidence to a potential role for SUA reduction in the management of diabetes [29–32]. A randomized controlled trial of 176 diabetic patients with hyperuricemia showed that the lowering uric acid was associated with ameliorative insulin resistance [29]. The similar result was also observed in a cohort study involving 73 participants with hyperuricemia [30]. However, the number of participants in these studies was not big enough to draw conclusions. And to our knowledge, no previous study has explored a possible role for SUA reduction in the prevention of diabetes. In our study, compared with a minor change of SUA level, a percentage SUA loss of ≥10% was associated with a 16% lower risk of developing diabetes after adjustment for important covariates. Our findings therefore imply that a reduction in SUA is associated with a decreased risk of diabetes. In terms of clinical significance, monitoring SUA regularly and reducing SUA may contribute to the prevention and delay of diabetes.

The progressive global epidemic of diabetes has resulted in diabetes being an important risk factor for adverse cardiovascular. Although our study suggests that a reduction in SUA is associated with a decreased risk of diabetes, it is unclear whether the reduction in SUA is caused by lifestyle modification or treatment with uric acid-lowering drugs. Further studies are needed to elucidate whether the effect of lowering SUA by lifestyle modification or treatment with uric acid-lowering drugs is different in preventing incident diabetes.

Our study has several strengths. It is the first to evaluate the relationship between changes in hyperuricemia and the risk of diabetes in Chinese urban adults, which can help to identify high-risk groups for diabetes. The second strength is that the study provide evidence for a potential role of SUA reduction in the prevention of diabetes. Finally, the prospective cohort study design and long follow-up period help the determination of the true relationship between changes in hyperuricemia and diabetes. However, several limitations need to be addressed. Firstly, some important risk factors for diabetes, such as nutritional factors, were not measured. Secondly, this study was conducted using a sample of the Beijing population, and therefore, our findings have limited generalizability to other populations. In fact, as reported elsewhere [33], it cannot be excluded that the genetic peculiarity in some populations may play different roles in this context. Thirdly, detailed information about medication use, especially uric acid-lowering drugs, was lacking in our study. Finally, as the information regarding lifestyle was self-reported, measurement errors are inevitable.

## 5. Conclusions

In summary, our findings show that changes in hyperuricemia, especially persistent hyperuricemia, are more appropriate to reflect the risk of diabetes than a single measure of SUA value at baseline. In addition, the study explores the potential role of SUA reduction in minimizing the future risk for developing diabetes in urban Chinese adults. Our study underscores the importance of SUA management and further suggests that attention should be paid to prevent hyperuricemia and maintain a healthy concentration of SUA to prevent diabetes.

## Figures and Tables

**Table 1:** Distribution of potential confounding factors of participants grouped by change of hyperuricemia from 2009 to 2011.

Baseline characteristic	Changes of hyperuricemia			
	No hyperuricemia	Remittent hyperuricemia	Incident hyperuricemia	Persistent hyperuricemia
Male (*n*, %)	5998 (50.42)	1022 (79.47)	683 (78.06)	1148 (85.29)^∗^
Age (years)	42.31 ± 13.59	45.01 ± 14.79	42.96 ± 15.07	46.88 ± 15.30^∗^
BMI (kg/m^2^)	23.81 ± 3.34	26.06 ± 3.33	25.91 ± 3.31	26.8 ± 3.05^∗^
SBP (mm Hg)	114.86 ± 14.80	121.44 ± 14.15	121.73 ± 15.12	123.21 ± 14.31^∗^
DBP (mm Hg)	75.34 ± 9.08	79.51 ± 9.16	79.78 ± 9.92	80.73 ± 9.32^∗^
TC (mmol/L)	4.83 ± 0.91	5.11 ± 0.99	4.96 ± 0.90	5.22 ± 0.98^∗^
TG (mmol/L)	1.27 ± 0.99	1.99 ± 1.65	1.77 ± 1.23	2.28 ± 1.84^∗^
HDLC (mmol/L)	1.38 ± 0.32	1.23 ± 0.29	1.23 ± 0.27	1.18 ± 0.25^∗^
High school or higher education (*n*, %)	11732 (98.62)	1274 (99.07)	864 (98.74)	1330 (98.81)
Physical activity (*n*, %)				
Low	1023 (8.60)	73 (5.68)	54 (6.17)	74 (5.50)^∗^
Moderate	10513 (88.37)	1180 (91.76)	802 (91.66)	1240 (92.12)
High	360 (3.03)	33 (2.57)	19 (2.17)	32 (2.38)
Smoking status (*n*, %)	600 (5.04)	85 (6.61)	62 (7.09)	121 (8.99)^∗^
Drinking status (*n*, %)	1034 (8.69)	137 (10.65)	85 (9.71)	191 (14.19)^∗^
Family history of diabetes (%)	698 (5.87)	58 (4.51)	53 (6.06)	103 (7.65)^∗^

^∗^ *P* < 0.05. Data were presented as the mean ± standard deviation or number (%). BMI = body mass index; SBP = systolic blood pressure; DBP = diastolic blood pressure; TC = total cholesterol; TG = triglycerides; HDLC = high-density lipoprotein cholesterol.

**Table 2:** The association between changes of hyperuricemia and incident diabetes.

Changes of hyperuricemia	Total	Diabetes (*n*, %)	RR (95% CI)		
			Model 1^∗^	Model 2^†^	Model 3^‡^
No hyperuricemia	11896	467 (3.93)	1.00	1.00	1.00
Remittent hyperuricemia	1286	115 (8.94)	1.87 (1.53-2.29)	1.54 (1.26-1.89)	1.35 (1.10-1.64)
Incident hyperuricemia	875	78 (8.91)	1.98 (1.56-2.51)	1.65 (1.30-2.09)	1.48 (1.17-1.86)
Persistent hyperuricemia	1346	181 (13.45)	2.62 (2.20-3.12)	2.05 (1.71-2.46)	1.75 (1.47-2.08)

Data were relative risks (RRs) and 95% confidence intervals (CIs). Hyperuricemia was defined as serum uric acid level greater than 7 mg/dL in males and serum uric acid level greater than 6 mg/dL in females. ^∗^Adjusted for age and sex at baseline. ^†^Adjusted for variables in model 1 as well as education level, smoking, alcohol consumption, physical activity, family history of diabetes, and body mass index at baseline. ^‡^Adjusted for variables in model 2 as well as systolic blood pressure, diastolic blood pressure, total cholesterol, triglycerides, and high-density lipoprotein cholesterol at baseline.

**Table 3:** Sensitivity analysis in the association between changes of hyperuricemia and incident diabetes.

Changes of hyperuricemia	Total	Diabetes (*n*, %)	RR (95% CI)		
			Model 1^∗^	Model 2^†^	Model 3^‡^
No hyperuricemia	11782	465 (3.95)	1.00	1.00	1.00
Remittent hyperuricemia	1182	100 (8.46)	1.70 (1.36-2.11)	1.42 (1.13-1.78)	1.25 (1.00-1.55)
Incident hyperuricemia	830	75 (9.04)	1.98 (1.54-2.53)	1.70 (1.33-2.18)	1.51 (1.18-1.93)
Persistent hyperuricemia	1609	201 (12.49)	2.42 (2.03-2.89)	1.93 (1.61-2.32)	1.65 (1.38-1.96)

Data were relative risks (RRs) and 95% confidence intervals (CIs). Hyperuricemia was defined as serum uric acid level greater than 6.8 mg/dL. ^∗^Adjusted for age and sex at baseline. ^†^Adjusted for variables in model 1 as well as education level, smoking, alcohol consumption, physical activity, family history of diabetes, and body mass index at baseline. ^‡^Adjusted for variables in model 2 as well as systolic blood pressure, diastolic blood pressure, total cholesterol, triglycerides, and high-density lipoprotein cholesterol at baseline.

**Table 4:** The association between percentage change of serum uric acid and incident diabetes.

Percentage change of serum uric acid	Total	Diabetes (*n*, %)	RR (95% CI)		
			Model 1^∗^	Model 2^†^	Model 3^‡^
≤ −10%	4133	223 (5.40)	0.81 (0.69-0.95)	0.83 (0.71-0.99)	0.84 (0.72-0.99)
> −10% and ≤10%	7663	412 (5.38)	1.00	1.00	1.00
>10% and ≤30%	2900	163 (5.62)	1.40 (1.17-1.68)	1.34 (1.12-1.60)	1.30 (1.08-1.55)
>30%	707	43 (6.08)	2.00 (1.48-2.72)	1.85 (1.37-2.50)	1.71 (1.27-2.30)
*P* for trend			<0.0001	<0.0001	<0.0001

Data were relative risks (RRs) and 95% confidence intervals (CIs). ^∗^Adjusted for age, sex, and serum uric acid at baseline. ^†^Adjusted for variables in model 1 as well as education level, smoking, alcohol consumption, physical activity, family history of diabetes, and body mass index at baseline. ^‡^Adjusted for variables in model 2 as well as systolic blood pressure, diastolic blood pressure, total cholesterol, triglycerides, and high-density lipoprotein cholesterol at baseline.

**Table 5:** The association between baseline hyperuricemia and incident diabetes.

Changes of hyperuricemia	Total	Diabetes (*n*, %)	RR (95% CI)		
			Model 1^∗^	Model 2^†^	Model 3^‡^
No hyperuricemia	12771	545 (4.27)	1.00	1.00	1.00
Hyperuricemia	2632	296 (11.25)	2.08 (1.81-2.41)	1.70 (1.46-1.98)	1.48 (1.28-1.71)

Data were relative risks (RRs) and 95% confidence intervals (CIs). ^∗^Adjusted for age and sex at baseline. ^†^Adjusted for variables in model 1 as well as education level, smoking, alcohol consumption, physical activity, family history of diabetes, and body mass index at baseline. ^‡^Adjusted for variables in model 2 as well as systolic blood pressure, diastolic blood pressure, total cholesterol, triglycerides, and high-density lipoprotein cholesterol at baseline.

## Data Availability

The data that support the findings of this study are available from the Beijing Health Management Cohort (BHMC) study, but restrictions apply to the availability of these data, which were used under license for the current study and so are not publicly available. Data are however available from the authors upon reasonable request and with permission of the Capital Medical University.
